# Sleep and Children with Cerebral Palsy: A Review of Current Evidence and Environmental Non-Pharmacological Interventions

**DOI:** 10.3390/children2010078

**Published:** 2015-02-27

**Authors:** Risha Dutt, Mary Roduta-Roberts, Cary A. Brown

**Affiliations:** Faculty of Rehabilitation Medicine, University of Alberta, 2-64 Corbett Hall, Edmonton, AB T6G2G4, Canada; E-Mails: rdutt@ualberta.ca (R.D.); mroberts@ualberta.ca (M.R.-R.)

**Keywords:** sleep, children, cerebral palsy, environment, light, sound, bedding, temperature

## Abstract

Between 23%–46% of children with cerebral palsy experience sleep problems. Many of the sensory-motor and cognitive features of cerebral palsy (such as immobility, pain, and seizures) act as predisposing factors for sleep problems in this population. This paper presents the background related to the etiology and consequences of sleep problems in children with cerebral palsy. The relationship between pain and sleep is emphasized, as the risk of pain is highly prevalent in children with cerebral palsy. The review concludes with a discussion of the evidence-base for environmental non-pharmacological interventions based on light, temperature, sound and bedding to promote sleep for children with cerebral palsy.

## 1. Introduction

Studies show that over the last 20 years sleep duration in children has declined [1] and the incidence of diagnosed sleep disorders is increasing. The prevalence of sleep problems is estimated to be 20% to 42% across childhood [2]. This is concerning given that sleep is foundational to wellbeing and healthy neurophysiologic functioning, learning and memory [3]. Sleep and sleep behaviours are regulated and modified by an extensive combination of factors, including genetic, biological, environmental, and behavioral influences [1]. Sleep problems have a wide range of causes and this paper focuses on the more common outcome of various sleep problems—sleep deficiency (SD). Sleep deficiency (SD) is a broad term defined by the American National Heart, Lung and Blood Institute (http://www.nhlbi.nih.gov/health/health-topics/topics/sdd) as insufficiency sleep or sleep patterns that interfere with physical and mental well-being. Sleep deficiency in children increases the risk of negative mood, behavioral problems (such as aggression), cognitive impairments, emotional problems (such as depression), poorer school performance and health problems (such as obesity and sensory processing impairments) [1,4]. These cognitive, emotional and physical health problems can, in turn, increase the likelihood of poor sleep performance such that a vicious cycle of increasing SD, poor health and decreased function becomes established.

Sleep deficiency (SD) in children with underlying neurodevelopmental disabilities is more difficult to identify and address than in typically developing children [4]. This paper will review the current SD evidence as it relates to one of the most common childhood disabilities—cerebral palsy [5]. Cerebral palsy is defined as a group of non-progressive disorders of movement and posture. These children are at higher risk for sleep problems because of the pain, mobility impairment, and sensory processing (integration and interpretation of sensory input) problems that often accompany cerebral palsy [5]. Sleep deficiency in children with cerebral palsy significantly affects the child’s physical, emotional and cognitive development and performance, and has been reported to affect caregivers and family who themselves can become sleep deprived [1,6]. Particularly, depression and other health issues have been noted in mothers of children with cerebral palsy and SD because of the disruptive impact that nighttime monitoring and care needs of a child with cerebral palsy can have on parents’ and siblings’ sleep and wellbeing [5]. When placed in this broader family system context it is clear that a child’s sleep problems need to be recognized as being of wider concern because of the physical and emotional toll that can result for the entire family. Although the evidence is clear that the negative impact of SD on a child with cerebral palsy and on their family can be extensive, Jan *et al.* [4] emphasize that there is a lack of applied clinical research and that redressing this gap is critically important.

### Objective

Children with cerebral palsy and their families encounter a wide range of healthcare practitioners and each profession’s knowledge-base about sleep and its impact on function and well-being can vary greatly. It is important for professionals who are in most frequent contact with these patients, such as rehabilitation therapists and educational psychologists, to have some basic knowledge of sleep issues related to cerebral palsy so they can recognize red flags and refer to specialists within the pediatric treatment team. The goal of this review is to raise awareness of the significance of SD for a large number of children with cerebral palsy and for their families. Readers unfamiliar with the types and degrees of severity of cerebral palsy are referred to the excellent reviews provided by Bax and Gillberg [7] and Stores [8]. This general review is not intended to imply that all children with cerebral palsy will experience some form of sleep problem. As such it focuses only on selected aspects, primarily the relationship between the aspects of the bedroom environment and sleep, which, in the authors’ experience, are most often overlooked.

Although many recent advances have been made in sleep research involving children with developmental delays this knowledge is not uniformly integrated into practice. This paper seeks to decrease the research-to-practice gap by summarizing the etiology of selected sleep problems (with particular focus on the relationship between sleep and pain in cerebral palsy), and provides a review of selected literature on evidence-based non-pharmacological sleep interventions (NPSI) that all healthcare practitioners who work with these children can incorporate into their recommendations to parents and caregivers.

## 2. Sleep Deficiency in Children with Cerebral Palsy

Cerebral palsy is one of the most common disorders in children and occurs at a rate of 2 to 2.5 per 1000 live births [4]. “Cerebral” refers to the cerebrum and “palsy” means disorder in movements. Therefore, in cerebral palsy, movement abnormalities result from cerebral damage and, consequently, childhood musculoskeletal problems are common [9]. Although presentation of cerebral palsy is quite varied a recent US study reported that approximately 48% of children having some form of mobility problem [10]. Cerebral palsy is characterized by an absence or delay in motor development, abnormal muscle tone, contractures, muscle tightness, deformities, and persistence of primitive and other body reflexes [9]. In turn, persisting body reflexes interfere with development of gross and fine motor movement. An additional feature of cerebral palsy is the high incidence of physiological problems such as gastro-esophageal reflux disorder (GERD). Chambers [9] reports that upwards of 80%–90% of children with severe cerebral palsy will also have chronic gastrointestinal issues of which gastro-esophageal reflux is the most common condition. Apart from musculoskeletal and physiological problems, other conditions associated with cerebral palsy include epilepsy, visual impairment, respiratory problems [5], drooling and uncoordinated swallowing [9]. All of these problems can impact a child’s sleep and will be discussed in greater depth in later sections. For a fully detailed review of the types of cerebral palsy and the range of severity readers are referred to the comprehensive work of Bax and Gillberg [7].

Sleep disturbance is reported to be very common in children with cerebral palsy and between 23% to 46% of children with cerebral palsy suffer from sleep problems [9,11]. This is significantly higher than that reported for typically developing children (20%–30%) [12]. Sleep problems experienced by children with cerebral palsy include: difficulty in initiating and maintaining sleep, sleep wake transition, sleep breathing disorders, sleep bruxism, excessive day time sleeping, nightmares and sleep talking [9,11]. In Elsayed’s study [13], involving 100 children with cerebral palsy divided into preschool and school-age group, both groups showed a high incidence of sleep problems. In the preschool group 46.2% experienced early insomnia and 50% demonstrated sleep bruxism (teeth grinding). In the school-age group 50% experienced sleep disordered breathing, 50% had nightmares, 12.5% regularly talked in their sleep, and 62.5% had excessive daytime sleeping. A high incidence of awakening after sleep onset (50%) in children with cerebral palsy was also found in a study by Pruitt and Tsai [14].

As mentioned previously sleep not only affects the overall development of the child, but also affects the well-being of the entire family. As Wayte *et al*. [5] reports, nearly 40% of children with cerebral palsy required parental attention on at least one occasion every night. In the same study 74% of parents reported that because of their child’s SD, their own daytime functioning was impaired.

### 2.1. Etiology

As previously mentioned, children with cerebral palsy are at high risk for sleep problems because of the numerous negative consequences of the condition (for example pain, spasticity, epilepsy and seizures). Epilepsy, for example, is a co-occurring condition in approximately 41% of children with cerebral palsy [10], and is associated with disturbed sleep. Children on antiepileptic drugs can also suffer from excessive daytime sleepiness [11]. Some children with cerebral palsy are also vulnerable to respiratory problems such as upper airway obstruction, which in turn can lead to repeated arousal from sleep [5]. Glossoptosis (abnormal downward or back placement of the tongue) and recurrent aspiration pneumonia related to gastro-esophageal reflux are also features of cerebral palsy in children with high level involvement and this can also induce sleep related breathing disturbance [11].

Newman’s review [11] of sleep and cerebral palsy also reported that disorders of initiation and maintenance of sleep were strongly associated the presence of visual impairment because abnormal light perception affects the regulation of sleep-related hormones such as melatonin and adenosine. Lastly, the review noted the significance of environmental factors (such as light, temperature, sound, and bedding) as contributors to the increased prevalence of sleep problems in children with cerebral palsy.

While there are many factors related to cerebral palsy that can interfere with sleep, pain is one factor that is common in children with cerebral palsy and is known to be underreported and undertreated in pediatric populations. For these reasons the remainder of this section will focus on the relationship between pain and sleep in children with cerebral palsy.

### 2.2. Relationship between Sleep and Pain in Cerebral Palsy

Pain is known to interfere with sleep and there is evidence that the relationship may be bi-directional such that sleep deprivation increases the severity of pain experience and increased pain interferes with sleep [15]. The risk of pain is highly prevalent in children with cerebral palsy and warrants particular attention in discussing sleep problems. Factors such as skin breakdown and pressure ulcers [5], spasticity, abnormal muscle tone, involuntary movements, and abnormal postures in children with cerebral palsy, can decrease their ability to change body position during nighttime and increases their experience of pain. In combination, these factors act as predisposing influences for sleep problems [11]. Ramstad, *et al.* [16] surveyed 153 participants with cerebral palsy aged 8 to 18 years and their parents. They found that 65% of the children reported moderate pain. Interestingly, parental reports of pain and perceived impact of pain on sleep were even higher than the children’s reports.

Research specific to pain and cerebral palsy is limited but what does exist seems to indicate that the two are closely related such that they may indeed have a bi-directional relationship. As a recent editorial in Developmental Medicine and Child Neurology about under-addressed issues for managing cerebral palsy points out “…pain is the most frequent problem [and] it is surprising how little attention it receives” [17]. Baxter cites the systematic review by Novak *et al.* [18] to support his position. The review appraised 82 research papers and concluded, among other things, that high level evidence existed demonstrating that three in four children with cerebral palsy experienced pain. Coupled with the high incidence of SD in children with cerebral palsy Novak *et al.* concluded that there is an “under-researched link between chronic pain, sleep and behavioural problems” and that this relationship “warrants urgent attention” [18]. Pruitt and Tsai [14] also reviewed the cerebral palsy literature and concluded that possible major causes of pain include musculoskeletal pain (such as hip dislocation or scoliosis), neuromuscular pain (such as muscle spasm), and gastrointestinal pain (such as gastro-esophageal reflux and constipation). They identified additional, less common, and potentially overlooked causes of pain including dental problems (such as abscesses), ophthalmologic problems (such as corneal abrasions), and urologic problems (such as bladder spasm).

In a study conducted by Breau and Camfield [19] 123 children with cerebral palsy were divided into three groups; those having no pain, those with treated pain, and those with untreated pain. Results indicated that children who had pain also had significantly more sleep problems overall, more night awaking, parasomnia, sleep disordered breathing and shorter sleep duration. Engel *et al.* [20] had similar findings in a study of 20 youths with cerebral palsy between the ages of 6 and 17 years, where the results indicated that pain interfered with participants’ self-care and with sleep.

Sleep disturbance in children with persistent pain may also be associated with underlying disease-related mechanisms (such as muscle spasm, or contractures), and treatment regimens (such as analgesics and antiepileptic sleep-disrupting medication). Emerging research seems to indicate that insufficient sleep contributes to increased pain sensitivity and dysregulation of the hypothalamic pituitary adrenal axis (HTPA). Evidence is accumulating that supports the existence of a bi-directional relationship between pain and sleep such that problems in one increase the risk of problems with the other [21,22].

## 3. Physiology of Sleep and Effect of Environmental Factors

Jin, Hanley and Beaulieu [23] determined that medical residents in the United Kingdom receive no more than 5 hours of sleep-related education. As such, their knowledge base can be limited. They concluded that it is therefore unsurprising that parents of children with cerebral palsy most often receive only pharmacological intervention despite the existence of evidence identifying side-effects and the limited evidence-base regarding long-term tolerability and efficiency of most pharmacological sleep interventions used in pediatric populations [23]. The need for non-pharmacological interventions (such as cognitive behavioural, activity-based, environmental and sleep hygiene interventions) is evident. There is a small but growing volume of literature exploring how environmental features in the home affect sleep. For example, types of bedding material, window coverings, artificial blue spectrum bright-light exposure at night (from televisions, computers and other electronic devices for example), nighttime household noises, and bedroom temperatures, all have a demonstrated physiological base for consideration in interventions to promote sleep. In keeping with the objective of this paper to present evidence-based knowledge that can be integrated into all healthcare providers’ interventions and recommendations, regardless of their professional background, and, because of the clear influence environmental features exert on sleep physiology, the remainder of this paper will focus more on environmental modifications and their particular relevance for children’s sleep. This is not intended to imply that other interventions (as listed above) are less effective. However, pragmatic, effective, sleep-promoting changes to the bedroom environment that should form the foundation to support any other sleep intervention can often be overlooked. This review will now focus on providing a general review of the evidence-base of the physiological relationship between sleep and light, sound, temperature and allergens.

Sleep is generated by the activity of specific brain structures (such as the superchiasmic nucleus and hypothalamus), neurochemicals and neural networks [24]. There are four stages to a sleep cycle that repeat throughout the night in a pattern of Stages 1 through 3, categorized as “non-rapid eye movement” NREM, leading to Stage 4, categorized as “rapid eye movement” (REM). Each cycle of all 4 stages is of approximately 90-min duration [3]. Stage 1 is light sleep, where eye movement and muscle activity slows. In Stage 2, eye movement stops and neural activity become slower; with an occasional burst of oscillatory brain activity, generated in the thalamus, called sleep spindles. In Stage 3 (called “deep sleep”) extremely slow brain waves (called “delta waves”) begin to appear and it is usually difficult to wake the sleeper. Stage 4 sleep consists of REM sleep and is characterized by high levels of neuronal activity. This stage is most commonly associated with dreaming [3].

There are two main processes that affect the sleep-wake cycle. Firstly, the primary sleep-inducing neurotransmitter adenosine accumulates during the day leading to activation of the areas of the hypothalamus creating a homeostatic drive for sleep [25]. Secondly, the suprachiasmatic nucleus in the hypothalamus (which is considered to be the control centre of autonomic functions such as thermoregulation), receives direct blue spectrum light input from the retina via the retinohypothalamic tract and this mechanism coordinates circadian rhythm [24]. Circadian rhythm is largely regulated by the light/dark cycle and, to a lesser degree, temperature [3].

### 3.1. Light

Light directly impacts human physiology and behavior through its alerting effects on circadian rhythm [26].The hormone melatonin is regulated in the hypothalamus and plays a central role in synchronization of circadian rhythm and especially the sleep/wake cycle. Bright blue spectrum light inhibits melatonin secretion during the day, and lower light levels (dusk) increase melatonin secretion in the evening and night [27]. Excess exposure to artificial blue spectrum light, usually through electric room lighting and electronic devices, will alter the timing, duration and amount of melatonin synthesis [28]. Exposure to blue spectrum light of as little as 50 lux has been shown to inhibit melatonin secretion [29] and researchers consistently recommend that the bedroom should be as dark as possible to promote sleep onset and maintenance. The increasingly prevalent practices of providing a television in children’s bedrooms, allowing the use of electronic mobile devices at night, and using electronic devices for bedtime story telling and games are therefore of concern. These electronic screens emit varying degrees of blue spectrum light and can lead to disrupted melatonin production which, in turn, greatly delays sleep onset [30].

In their comprehensive review of infants’ sleep physiology Heraghty *et al.* [3] noted evidence suggesting that infants who are exposed to constant illumination in the nursery face more problems with circadian rhythm as compared to those who are exposed to dim light. It has been suggested that the pattern of light/dark exposure has an even greater influence on a child’s circadian rhythm than does a fixed sleep schedule [31]. Therefore both daytime blue spectrum light exposure and also controlling evening blue spectrum light exposure can be manipulated to promote sleep onset and maintenance. Careful consideration of frequency and timing of blue spectrum light is particularly important for children with cerebral palsy who may not have the same opportunities and abilities to participate in outdoor e activities required to receive sufficient daytime blue spectrum light exposure.

### 3.2. Sound

Environmental noise is one of the risk factors that influences sleep/wake behavior and sleep quality [32]. Environmental noise is defined as unwanted or harmful sound in the home environment that can be caused by human activity (for example running machinery, conversation, and also outdoor activities such as that produced by vehicles). Sound has an influence on the central nervous system and on various autonomic functions, such that stress hormones are released. For example, the stress hormone cortisol is produced as a response to alerting night time noises, and leads to autonomic reactions such as increased heart rate, blood pressure, respiratory rate and muscle tension [32]. Increased production of cortisol is also of significance because in well-rested individuals cortisol levels drop over the nighttime. This means that elevated cortisol levels on wakening indicate a cause for concern. The effect of noise on sleep includes; a delay in sleep onset, inability to move into the deeper stages of sleep, more frequent awakenings (and thereby interrupted sleep cycles), increased restlessness and body movements, and an overall shortening of total sleep time. To-date there is a paucity of research directly related to children with cerebral palsy and noise. However, lessons from other studies can be extrapolated and the review by Jan *et al.* [4] concluded that slight modifications in the bedroom, the use of ear plugs, or the use of “white noise” machines (which produce a mixture of all frequencies and can mask discrete alerting sounds) may be of benefit.

### 3.3. Temperature

Ambient temperature has an important influence on sleep because thermoregulation affects circadian rhythm and other sleep governing mechanisms [33]. Normally, a slight drop in core temperature occurs when the homeostatic drive to sleep is high and so the likelihood of sleep initiation increases. High ambient temperatures interfere with the body’s ability to achieve a drop in core temperature and so slightly cooler bedroom temperatures are recommended. Core body temperature continues to decreases slightly through the night, and particularly during REM stage sleep when skin temperature in the peripheral extremities is at its lowest. The amount of time spent in REM stage sleep increases over the night and most REM sleep occurs in the 2–3 h prior to wakening. Children with cerebral palsy and motor impairments may require the bedroom ambient temperature to be increased during this period because the thermoregulatory responses of shivering and body movements are suppressed during REM stage sleep [33]. A recommended ambient temperature for sleeping is between 18–22 degrees Celsius [33]. A programmable thermostat set for lower temperatures at bedtime and then increased temperature two hours before wakening can help create an optimum sleep environment for these fluctuating needs.

### 3.4. Bedding

There is little evidence-based research regarding the impact of the sleeping surface and bedding on the sleep quality of children with neurodevelopmental disability [4]. However, choice of mattress, pillow, sheets and blankets may be important for some children for both comfort and for allergen reduction. Children with decreased motor control, or inability to independently reposition themselves, can benefit from environmental modifications (such as the use of programmable thermostats) to create sleep conducive atmospheres. Certain types of bedding are more likely to trigger wheezing, and particularly wheezing caused by house dust mites (HDM) related airway obstruction [34]. Synthetic quilts, pillows, and electric blankets are very commonly in use these days, and these have been associated with frequent breathing problems during sleep in children. Some research suggests this is because higher counts of HDMs and allergens occur in synthetic quilts and pillows as compared with feather duvets and pillows that have tight cotton ticking that reduces the ability of HDM and particles to be released near the child’s airway [34]. All forms of bedding, including mattresses, pillows, duvets, bedding and under-bedding can be significant HDM and allergen reservoirs [34]. Allergen barrier underbedding and pillow covers, frequent washing of bedding, putting pillows and duvets through the dryer, and frequent vacuuming of mattresses can help reduce the risk exposure.

Children with neurodevelopmental disability and sensory processing problems (such as tactile defensiveness) also may have a specific preference for either light or heavy blankets [4,35]. Children who has sensory processing (or sensory interpretation) problem can, for example, find a light blanket painful or very distressing to have in contact with their skin. In these situations they try to avoid the blanket; hence the term, “tactile defensiveness”. Use of sleep products such as supracor^®^ sheets (http://www.supracor.com/company/), which are made of a flexible, breathable, honeycomb-like, absorbent material, may help minimize the need to change pajamas and bedding which, in turn, may result in less sleep disruption for both the child and caregivers.

## 4. Evidence-Based Non-Pharmacological Intervention

Non-pharmacological sleep interventions (NPSI) are important in addressing sleep problems and are recommended as adjunctive or alternatives to long-term use of sleep medication [36,37,38]. Such sleep interventions include; therapeutic use of activity to develop a sleep routine with a regular bedtime and calming sleep behaviors, environmental modification in the home (such as light, temperature, bedding and sound), and interventions designed to improve parental sleep knowledge and ability to problem solve. Applied research is sparse and the reader is referred to a recent critical review of NPSI for youth with chronic health conditions which found that the methodological quality of the few existing studies of NPSIs for this population was low [38]. However for certain interventions (specifically bright light therapy, activity, massage and behavioural interventions) the reported outcomes of studies in the review were consistently positive and with no adverse effects. The authors concluded these interventions were promising and warranted more rigorously designed and contextually relevant study.

## 5. Conclusions

Research demonstrates that sleep deficiency is a common consequence with significant negative impact for children with cerebral palsy. Not only are sleep deprived children at risk of developing or exacerbating other physical and emotional health problems but so are other members of the family, who themselves become sleep deprived consequent to caregiving roles and additional family pressures. Knowledge of basic sleep physiology, and how to apply this knowledge to make non-pharmacological, sleep environment recommendations to parents, will be of benefit to healthcare providers from all disciplines. There is a clear gap in the application of the existing light, temperature, sound and bedding evidence-base. Studies of parent and healthcare professional knowledge translation of NPSIs (particularly those focusing on environmental modifications) are warranted.

## References

[1] Magee C.A., Caputi P., Iverson D.C. (2012). Are parents’ working patterns associated with their child’s sleep? An analysis of dual-parent families in Australia. Sleep Biol. Rhythms.

[2] Bharti B., Mehta A., Malhi P. (2013). Sleep problems in children: A guide for primary care physicians. Indian J. Pediatr..

[3] Heraghty J., Hilliard T., Henderson A., Fleming P. (2008). The physiology of sleep in infants. Arch. Dis. Child..

[4] Jan M.M. (2006). Cerebral palsy: Comprehensive review and update. Ann. Saudi Med..

[5] Wayte S., McCaughey E., Holley S., Annaz D., Hill C.M. (2012). Sleep problems in children with cerebral palsy and their relationship with maternal sleep and depression. Acta Paediatr..

[6] Simard-Tremblay E., Constantin E., Gruber R., Brouillette R.T., Shevell M. (2011). Sleep in children with cerebral palsy: A review. J. Child Neurol..

[7] Bax M., Gillberg C. (2010). Comorbidities in Developmental Disorders.

[8] Stores G. (2014). Sleep and Its Disorders in Children and Adolescents with a Neurodevelopmental Disorder.

[9] Chambers H.G. (2002). Advances in cerebral palsy. Curr. Opin. Orthop..

[10] Christensen D., van Naarden Braun K., Doernberg N.S., Maenner M.J., Arneson C.L., Durkin M.S., Benedict R.E., Kirby R.S., Wingate M.S., Fitzgerald R. (2014). Prevalence of cerebral palsy, co-occurring autism spectrum disorders, and motor functioning—Autism and Developmental Disabilities Monitoring Network, USA, 2008. Dev. Med. Child Neurol..

[11] Newman C.J., O’Regan M., Hensey O. (2006). Sleep disorders in children with cerebral palsy. Dev. Med. Child Neurol..

[12] Tikotzky L., Sadeh A. (2001). Sleep patterns and sleep disruptions in kindergarten children. J. Clin. Child Psychol..

[13] Elsayed R., Hasanein B., Sayyah H., El-Auoty M., Tharwat N., Belal T. (2013). Sleep assessment of children with cerebral palsy: Using validated sleep questionnaire. Ann. Indian Acad. Neurol..

[14] Pruitt D.W., Tsai T. (2009). Common medical comorbidities associated with cerebral palsy. Phys. Med. Rehabil. Clin. N. Am..

[15] Schuh-Hofer S., Wodarski R., Pfau D.B., Caspani O., Magerl W., Kennedy J.D., Treede R. (2013). One night of total sleep deprivation promotes a state of generalized hyperalgesia: A surrogate pain model to study the relationship of insomnia and pain. Pain.

[16] Ramstad K., Jahnsen R., Skjeldal O., Diseth T.H. (2011). Characteristics of recurrent musculoskeletal pain in children with cerebral palsy aged 8 to 18 years. Dev. Med. Child Neurol..

[17] Baxter P. (2013). Comorbidities of cerebral palsy need more emphasis—Especially pain. Dev. Med. Child Neurol..

[18] Novak I., Hines M., Goldsmith S., Barclay R. (2012). Clinical prognostic messages from a systematic review on cerebral palsy. Pediatrics.

[19] Breau L.M., Camfield C.S. (2011). Pain disrupts sleep in children and youth with intellectual and developmental disabilities. Res. Dev. Disabil..

[20] Engel J.M., Petrina T.J., Dudgeon B.J., McKearnan K.A. (2005). Cerebral palsy and chronic pain: A descriptive study of children and adolescents. Phys. Occup. Ther. Pediatr..

[21] Goodin B.R., Smith M.T., Quinn N.B., King C.D., McGuire H. (2012). Poor sleep quality and exaggerated salivary cortisol reactivity to the cold pressor task predict greater acute pain severity in a non-clinical sample. Biol. Psychol..

[22] Valrie C.R., Bromberg M.H., Palermo T., Schanberg L.E. (2013). A systematic review of sleep in pediatric pain populations. J. Dev. Behav. Pediatr..

[23] Jin C.S., Hanley G.P., Beaulieu L. (2013). An individualized and comprehensive approach to treating sleep problems in young children. J. Appl. Behav. Anal..

[24] Moszczynski A., Murray B.J. (2012). Neurobiological aspects of sleep physiology. Neurol. Clin..

[25] Evers S. (2010). Sleep and headache: The biological basis. Headache.

[26] Chellappa S.L., Steiner R., Oelhafen P., Lang D., Götz T., Krebs J., Cajochen C. (2013). Acute exposure to evening blue-enriched light impacts on human sleep. J. Sleep Res..

[27] Van Maanen A., Meijer A.M., Smits M.G., Oort F.J. (2011). Termination of short term melatonin treatment in children with delayed dim light melatonin onset: Effects on sleep, health, behavior problems, and parenting stress. Sleep Med..

[28] Gooley J., Chamberlain K., Smith K., Khalsa S., Rajaratnam S., van Reen E., Lockley S. (2011). Exposure to Room Light before Bedtime Suppresses Melatonin Onset and Shortens Melatonin Duration in Humans. J. Clin. Endocrinol. Metab..

[29] Wood B., Rea M.S., Plitnick B., Figueiro M.G. (2013). Light level and duration of exposure determine the impact of self-luminous tablets on melatonin suppression. Appl. Ergon..

[30] Garrison M., Liekweg K., Christakis D.A. (2011). Media use and child sleep: The impact of content, timing, and environment. Pediatrics.

[31] Appleman K., Figueiro M.G., Rea M.S. (2013). Controlling light-dark exposure patterns rather than sleep schedules determines circadian phase. Sleep Med..

[32] Van Kamp I., Gidlof-Gunnarsson A., Persson Waye K. (2013). The effects of noise disturbed sleep on children’s health and cognitive development. J. Acoust. Soc. Am..

[33] Okamoto-Mizuno K., Mizuno K. (2012). Effects of thermal environment on sleep and circadian rhythm. J. Physiol. Anthropol..

[34] Ponsonby A., Dwyer T., Trevillian L., Kemp A., Cochrane J., Couper D., Carmichael A. (2004). The bedding environment, sleep position, and frequent wheeze in childhood. Pediatrics.

[35] Dawson G., Watling R. (2000). Interventions to facilitate auditory, visual, and motor integration in autism: A review of the evidence. J. Autism Dev. Disord..

[36] De Niet G.J., Tiemens B.G., Kloos M.W., Hutschemaekers G.J.M. (2009). Review of systematic reviews about the efficacy of non-pharmacological interventions to improve sleep quality in insomnia. Int. J. Evid. Based Healthc..

[37] Heussler H., Chan P., Price A.M.H., Waters K., Davey M.J., Hiscock H. (2013). Pharmacological and non-pharmacological management of sleep disturbance in children: An Australian paediatric research network survey. Sleep Med..

[38] Brown C.A., Kuo M., Phillips L., Berry R., Tan M. (2013). Non-pharmacological sleep interventions for youth with chronic health conditions: A critical review of the methodological quality of the evidence. Disabil. Rehabil..

